# Role of digitalization in environment, social and governance, and sustainability: Review-based study for implications

**DOI:** 10.3389/fpsyg.2022.961057

**Published:** 2022-11-30

**Authors:** Jiaqi Xu, Shengxiang She, Wen Liu

**Affiliations:** ^1^School of Economics, Management and Law, Shaanxi University of Technology, Hanzhong, China; ^2^School of Economics and Management, Northwest University, Xi’an, China; ^3^School of Business Administration, Guizhou University of Finance and Economics, Guiyang, China

**Keywords:** digitalization, sustainable development, socio-economic development, environment, climatic change

## Abstract

Digitalization gives people access to a vast network of untapped data, which has the potential to help society and the environment. Smart systems connected to the internet can systematically provide a unique opportunity to solve difficulties related to long-term sustainability. The goals are to create an egalitarian, ecologically sustainable, and healthy society. Technological resources are envisioned as game-changing instruments. Three key concerns integration benefits are highlighted here: (i) sustainable development goals, (ii) socio-economic development, and (iii) the role of digital technology in environmental sustainability. This viewpoint describes the potential that digitization can create a future sustainable society. The technological network would unite the urban and rural worlds under a shared banner of sustainable development, keeping all social elements in the loop. Nations that take a comprehensive strategy will be able to provide equitable growth and an efficient, sustainable, and digital existence for their citizens. As a result, digitization provides better living conditions, active public involvement, clean governance, and transparency in public welfare programs and processes. People who are well-informed, self-aware, and digitally equipped will be better learners, thinkers, reformers, participators, and change and growth agents, marching forward on sustainable progress. The advantages of digitization in hastening the transition to sustainable industrial processes and improving people’s health and happiness are explored. Finally, the perspective encapsulates the advantages of digitization by offering a holistic vision of how technology could aid in addressing major challenges such as endangered world biodiversity and climate change.

## Introduction

Despite the advance and increasing technological and digital revolution seemingly advantageous for economic development, it has created its environmental footprint. According to Shift Project (2019), almost 3.7% of the world’s carbon pollution comes from increasing digitalization which has escalated by approximately 70% from the year 2013 to 2020. The process of digital transformation that has been growing in the globalized economy for several years is intimately tied to the concept of Industry 4.0 and is fueled by revolutionary digital technologies (Hizam-Hanafiah and Soomro, 2021). Recently, this approach has expanded to include practically every aspect of life. Particularly stressful transformations are occurring in industrial and service organizations, which, due to massive competitiveness, must swiftly adjust to the transformations connected with the economy’s digitization (Botha, 2018). To gain a competitive edge, these organizations are progressively using digital technologies linked with the concept of Industry 4.0 (Kamarul Bahrin et al., 2016), which is also highly affected by the open innovation model they are progressively employing (Skordoulis et al., 2020). This strategy leads to the creation of partnerships between individuals and organizations outside of a specific organization and allows highly effective information exchange. System Integration, Simulation, Augmented Reality, Cloud Computing, Big Data and Analytics, Cyber-Physical systems, Cybersecurity, Additive Manufacturing, and Collaborative Robotics (Rüßmann et al., 2015) are some of the key technologies associated with the concept of Industry 4.0 which support the digital economy development. The proliferation of these solutions and their rising accessibility urge businesses to swiftly acquaint themselves with them and assess the viability of using them (Sony and Naik, 2019). Despite the rising and readily accessible knowledge about the capabilities of current digital technologies and their appeal, enterprises often encounter implementation difficulties (Sanders et al., 2016). Organizations are under mounting stress to integrate digital technology to evolve and renew their marketing strategies. Recent studies reveal, however, that many are not prepared to adapt to digital changes (see Kane et al., 2015). Therefore, there seems to be a mismatch between market expectations and organizational response capabilities.

The world is entering a period of digitization, in which most of our everyday activities rely heavily on cutting-edge digital and computer technology. These modern technologies are used in socio-economic, environmental, sustainability, and climatic studies to improve a system’s production and efficiency (Balogun et al., 2020; Ceipek et al., 2021). The existing energy systems are predicted to become more sustainable due to digitalization, which will help extend electricity to more remote locations and improve how best to utilize it. However, digitalization may introduce new challenges to the present energy systems, which we must anticipate. The rise of internet-connected smart technologies opens new avenues for logically addressing difficulties related to the United Nations Sustainable Development Goals (SDGs), which aim to build an ecologically sustainable and healthy society. This point of view explains the benefits of digitization in building a sustainable future society.

Digitalization is a pressing necessity with enormous potential to alter economic growth parameters, forging a mutually beneficial relationship with employment generation and long-term sustainability. Digitalization has stretched its wings over all aspects of existence in this era of technological growth, where everything revolves around the digital world. It has evolved as a key tool for simplifying the functioning and procedures in numerous aspects of the socio-economic sphere, such as administration, regulation, planning, and operations, improving people’s quality of life. This aspect of the digital era contributes to long-term development. Civilizations that have been digitally empowered are more aware, connected, compliant, collaborative, and satisfied with their development. They contribute as credible resources for the future of this country in consideration.

Environmental, socio-economic, and governance criteria are standards of behavior used by politically progressive investors to assess potential investments. Environmental criteria examine a company’s environmental policies, specifically its climate change policy. The social criteria of the firm look at how it maintains relationships with its employees, suppliers, customers, and the communities in which it operates. The company’s ties with stakeholders are examined using social criteria. Governance includes the company’s leadership, CEO remuneration, audits, internal controls, and shareholder rights. Before research, a broader range of topics is required to inform investors, businesses, and regulators adequately. Environmental problems may include corporate climate policy, energy usage, wastage, contamination, resource conservation, and animal welfare. The criteria could also be used to evaluate any company’s potential environmental risks and how those risks are managed. Direct and indirect greenhouse gas emissions, hazardous waste management, and environmental legislation may all be factors to consider. Apart from the reported advantages of digitalization, one must not be certain about the real influence of digitalization on the three (Environmental, socio-economic and governance, and sustainability) sectors. Digital technologies rapidly disrupt today’s treasury function; many businesses discover that next-generation technology may help them achieve their environmental, socio-economic, and governance goals. Rather than competing, they can complement one other, assisting firms in achieving vital priorities that are becoming increasingly important to employees, constituents, and customers. The importance of digitalization motivated the study to crack the existing trend regarding its positive role in the environment, socio-economic, governance, and sustainability.

Following the backdrop, the first objective of this study is to analyze whether digitalization affects environmental quality. For that reason, digitalization has positive and negative impacts on the environment which the study tends to observe. Secondly, this study tends to analyze the existing literature for the true relationship between digitalization and social development and governance. Despite the fact, that digitalization affects the environmental quality but it also provides solutions for sustainability that are essential for economic development. The last objective of this study is to investigate the relationship between digitalization and sustainability from the prevailing literature. However, several studies have been provided in the literature that reveals the positive impact of digitalization on environmental sustainability, improved social development and governance, and sustainable development. Still, there is a lack of comprehensive analysis regarding the role of digitalization in the discussed sectors.

Based on the objectives, this study could contribute to the existing literature in three ways. Firstly, this study provides a comprehensive look into the influence of digitalization on the environment, social development, and sustainability. However, the existing literature is extensive in covering all three sectors. Still, the literature regarding digitalization mostly covers the developed region and is limited to a specific state, country, or region. However, this study provides a comprehensive review-based association between the variables under consideration. Secondly, this research aims to create a new institutional approach to how digitalization contributes to improved sustainable development and its role in the environment, society, and government, all of which have made significant progress in human existence. Lastly, this study provides a review of diverse literature, which helps in the construction of policies that could help scholars and governors to take appropriate steps regarding digitalization and its role in improving environmental quality, social development, governance, and development.

The following is how the paper is structured: In section “Introduction,” an introduction was given. The literature review is discussed in section “Literature review.” Section “Theoretical framework, hypothesis development, and methodology” provides the theoretical framework, hypothesis development, and Methodological setup. Section “Discussion” is further classified into three sub-sections, which discuss the aims of digitalization and long-term growth, society’s economic development and digitalization, and the significance of digital technology in environmental sustainability. Section “Conclusion and policy implications” has proceeded with the concluding remarks and policy implications.

## Literature review

People have heard about digitization, the social revolution brought about by the widespread use of digital technologies to generate, process, share, and transact information. Digitalization uses digital technology and channels to access, exchange, and utilize information cost-effectively, quickly, and easily. With numerous breakthroughs in digital technology, the boom in Information and Communications Technology use increased. Cell phones have evolved into smartphones,’ with over 5 billion users, while internet usage has increased to 3 billion since 2015. This epoch transformed the flow of information, ushering us into the digital age, in which we use a variety of platforms to access, share, create, and convey data (Digital Revolution, 2015). This signals the beginning of our journey from being connected to being well informed to being socially empowered, and as a result, we are now self-sufficient and future-ready as we march toward sustainable development.

We were all smacked by a huge wave of global transformation as we entered the year 2022. Because the flow of information is now ubiquitous, this revolution has made the world increasingly digital in all its acts and closed the gap between the oblivious and informed classes. Because of the power of digitization, everyone is internationally always connected through their mobile devices. Social growth has resulted from increased socialization, reducing the social gaps. According to certain estimates, 75% of the global population has been online since April 2022. This has increased by 100% in the last 7 years and is likely to continue to rise. With mobile services becoming increasingly popular, we will soon reach the 100% mark. All areas, such as social, economic, and administrative, are now covered by this, providing great potential for progress in developing countries. According to statistics, mobile phones now account for up to 80% of all market usage, and around 72% of the population in some developing countries, such as India, uses mobile phones to access online services. As a result of this expanding trend, the market has become more sensitive and digitally dynamic. A year-over-year user increase demonstrates our growing social media and digital transformation footprints.

To achieve these objectives, significant trade-offs must be made. Consider the case where huge data is unregulated. In that circumstance, societal hazards could arise, and possible environmental gains or costs would be susceptible to unpredictably high technical innovation rates and system dynamics. On the other hand, Unrestricted digitalization might provide first-mover advantages in developing new markets and lifestyles. According to new transition studies, big data can help standardize rules and speed up the transition process. Non-professionals are invited to participate in the production of knowledge and big data through digital citizen science (e.g., by collecting, classifying, and sharing auditory or visual signals of urban places recorded with their smartphones), or groups of activist individuals develop new knowledge without being invited (Dickel and Franzen, 2016). Citizen science programs are especially common in the environmental domain, where volunteers use mobile devices to participate (e.g., monitoring air, light, or water pollution at different locations). OpenStreetMap could be a fantastic illustration of how volunteered geographic data can be put to good use (Haklay, 2010). It’s a global, community-driven open-access database of street networks, buildings, and public utilities^1^. BBBike is an Open Street Map-based crowdsourcing initiative that provides free bike routing in Berlin and 200 other cities worldwide (Lenz and Heinrichs, 2017). In the reality of the developing field of citizen science, volunteers are far more likely than self-determined researchers to function as human sensors for data gathering, and it is vital to learn from both unsuccessful citizen science projects and successful examples, such as BBBike, to make citizen science relevant for the public governance of big data by empowering people. Increasing public understanding of big data is one example of this (Michael and Lupton, 2016).

Introducing new technology signals a shift in economic systems, not to mention their reputation for delivering socio-economic services as economic drivers. Although digitization is a fast-evolving sector of national concern, particularly regarding the economy’s long-term viability, it has both benefits and drawbacks, with scientists opposing comments. The search for variables impacting economic growth in the socio-economic sector has changed as research trends in the business sector have evolved. Along with societal advancement, rising industrialization, environmental deterioration, and other independent variables, the business sector is fast changing There is a genuine need to investigate the influence of various variables on economic growth in business in changing social and economic conditions (e.g., factors generating quick changes like COVID-19), and there is a real need to explore the impact of various factors on economic growth in business in changing social and economic conditions.

External consequences will be linked to three key pillars of sustainable development: the environmental, social, and economic (Zhao et al., 2018; Ziolo et al., 2019). CSR (corporate social responsibility) is an increasingly important concept for business firms and stakeholders. Corporate social responsibility is a strategic strategy business organizations use to build positive stakeholder and consumer impressions. Corporate social responsibility performance’s environmental, social, and governance elements may help tourist firms perform better financially. Stakeholders and customers care about environmental, social, and governance problems. Many studies show that businesses that include sustainability concepts in their business models may improve their social, environmental, and economic performance. From this perspective, there is plenty of room in the business industry for sustainability as a new source of economic growth. Table 1 shows research results on digitalization, sustainable development, and the economy.

**TABLE 1:** Literature review of digitalization, sustainable development, and economy.

No.	Author	Research direction	Results
1	Huang et al., 2016; Tiefenbeck et al., 2016; Kim and Hall, 2019	Virtual reality and Sharing economy	A positive association between digitalization and numerous elements impacting the tourist business.
2	Buhalis and Law, 2008	Relationship between economic growth and digital technology	Defining four tourist characteristics that can drive a variety of changes, most of which are tied to economic growth: Sector of employment, penetration rate, technology, and value factor
3	Gruber, 2017; Okhimenko et al., 2019	Influence of policy factors	Tourism digitization is a unique industrial policy goal; failing to fulfill this challenge could have far-reaching economic consequences.
4	Adamczewski, 2016; Dorofeyev et al., 2018	Effect of system stability and data	Data and system stability: Big Data could be used to improve the efficiency of financial services and institutions’ internal risk management and external monitoring.
5	Pshenichnikov, 2017; Afonasova et al., 2019	Financial services	The impact of financial services on the tourism industry’s expansion through digital technology
6	Rayna and Striukova, 2016	The impact of new technologies on business models	Despite the flexibility of operation, research on digitalization in the tourism industry suggests that efficiency increases with the quick introduction of changes, and the propensity to have a low-cost structure is enhanced. A hasty redefining of business models might result in disruptive activity to established paradigms.
7	Watanabe et al., 2018	Productivity in industrialized countries	The influence of digitalization on the tourist economy’s productivity is being investigated. Researchers wanted to know if there is a probable productivity paradox in the digital economy and if productivity in developed countries is declining.
8	Watkins et al., 2018	Measured international transactions and assets	Research into various measurement methodologies and elements that influence gross domestic product levels, the impact of digitalization on assessing international transactions and assets, and the scope of activities and services.
9	Mostafa et al., 2019	Digitalization limiting human intervention	According to research, limiting human interaction and connecting everything boosts productivity.
10	Graham et al., 2017	Influence of digital technology on economic and social transformation	Digital technology implementation in the tourism sector and industry might result in economic and social change.
11	Haseeb et al., 2019	Sustainable development	The influence of digitization on tourism and the economy’s long-term commercial performance
12	Noeh et al., 2022	Sustainable development, digital literacy, infrastructure, governance	digitalization that will enhance the quality of infrastructure, governance, institutions, and knowledge flow that will encourage sustainable development
13	Wekerle et al., 2022	Sustainable development,	digitalization not only enhances students’ learning capabilities but also increases coordination and communication that influence technology-driven knowledge sharing and decision making which is significant for sustainable development
14	Ren et al., 2022; Wei and Ullah, 2022	Environmental sustainability and development	Digitalization significantly promotes environmental quality due to improvement in green innovation and reduction in carbon emissions
15	Aleksandrova et al., 2022	Sustainable development, the transition toward digitalization	digitalization substantially replaces the traditional techniques of economic growth, improving productivity that leads to economic sustainability.

### Review of relevant studies on the nexus of digitalization and sustainable development, social development, and environment

Apart from the above discussion, the current literature covers several aspects of digitalization on sustainable development, social development, and the environment. Regarding the role of digitalization on economic growth and sustainable development, the scholarly literature is extensive. For instance, the recent studies of Irtyshcheva (2021), Aleksandrova et al. (2022), and Hosan et al. (2022) empirically asserted that digitalization substantially replaces the traditional techniques of economic growth as well as competitiveness, enhances the productivity of the industrial sector, trade, and other economic activities by conserving fewer resources and production of lesser pollution, which leads to sustainable development. However, digitalization varies from country to country, where the earlier European Union (EU) economies are performing better with diversity in digitalization, while the new EU economies are lagging in the field of digitalization (Brodny and Tutak, 2022a). Similarly, discrepancies also exist in the small and medium enterprises of the said region, which holds substantial disparities between the EU-27 and Eu14 economies (Brodny and Tutak, 2022b). Besides, the recent study by Noeh et al. (2022) and Ren et al. (2022) argued that it is essential for a state to promote digitalization that will enhance the quality of infrastructure, governance, institutions, knowledge flow, digital literacy, financial development, among others, which promote sustainable development of the region. Therefore, Novikova et al. (2022) reinforce the implementation of digitalization in a country to achieve its target of economic growth and sustainable development.

Regarding social development, the recent study by Lavicza et al. (2022) reveals that digitalization helps improve pedagogical and educational activities and makes these activities more effective. Specifically. Wekerle et al. (2022) argued that when the technologies are implemented in the classrooms of the students, relatively their constructive, passive, and active learning abilities improve from those with no technological implementation in the class. Besides, digitalization not only enhances students’ learning capabilities but also increases coordination and communication that influence technology-driven knowledge sharing and decision making. As a result, digitization leads to better job performance, creative thinking, and student innovation (Deng et al., 2022; Wannapiroon and Pimdee, 2022). Following these specifications of digitalization, the recent study by Shurygin et al. (2022) intimates the implementation of digital technologies (such as laptops, smartphones, tablets, etc.) should be implemented in the educational institution, where 74% of the study sample respondents agreed to its progressive role in learning abilities.

Concerning the environmental impact of digitalization, Maiurova et al. (2022) analyzed two cities in Germany (Berlin and Moscow) and claimed that digitalization is a revolutionary factor in reduced municipal solid wastes and greenhouse gas emissions, conservation of raw material, employment generation, and enhances the efficiency of energy and machinery. Where the existing literature reveals that traditional fossil fuel consumption is directly linked to enhanced emissions (Abumunshar et al., 2020). Still, financial development and digitalization help reduce the emissions level by promoting renewable energy generation and consumption (Maiurova et al., 2022; Samour et al., 2022). Besides, including digital technologies in the energy imaginaries could benefit the energy sector by enhancing its productivity (Strengers et al., 2022). Concerning the role of digitalization in environmental quality, several studies provide evidence in the recent literature. For instance, Garske et al. (2021), Chen (2022), Ha et al. (2022), Ma et al. (2022), Ren et al. (2022), and Wei and Ullah (2022) empirically reveals that enhancement in digitalization substantially improves environmental quality by reducing the level carbon, greenhouse gas, and other pollution emissions. Digitalization enhances technological innovation, which further boosts energy-efficient resource consumption, lowers energy (fossil fuel) demand, and consequently dampens emissions and other pollution levels. Besides, digitalization encourages green total factor energy efficiency (Gao et al., 2022), drives the export value of green goods (Ha and Thanh, 2022), promotes green globalization (Ramos-Meza et al., 2021), and declines the production scale of pollution-intensive enterprises (Wen et al., 2021). Hence, the economies adopting digitalization could achieve a sustainable environment. ‘The relevant studies regarding digital technology and its use is provided in Table 2.

**TABLE 2:** Overview of the role of digital technology and its use.

No.	Digital technology concept	Role and procedure	References
1	Green Digital technology	Digital technology is used to power energy-efficient equipment and systems that promote energy efficiency in business processes.	Chou and Chou, 2012
2	Digital marketing and sustainable development	Efforts are made throughout the life cycle of digital technology and digital technology-enabled products and services, including design, production, application, operation, and disposal, to reduce the bad effects and maximize the positive benefits of human behavior on the environment.	Elliot, 2011
3	The environmental problems of digitalization	“First-order effects directly impact information technology gear throughout its lifecycle, including manufacturing, use, and disposal.”	Dedrick, 2010
4	Digital marketing that is environmentally sustainable	“ICTs have second-order effects on other systems like transportation and industrial production, altering their environmental implications.” Effects of a third-order “When the widespread use of ICTs leads to changes in people’s lives and economic systems, this happens.	Bose and Luo, 2011
5	Green Digital technology	Computer science is the study and practice of efficiently and successfully developing, producing, utilizing, and disposing of computers, servers, and other subsystems such as displays, printers, storage devices, and networking and communications systems.	Dedrick, 2010; Butler, 2011
6	Green Digital technology	’The technique of efficiently and successfully designing, producing, and using computers, servers, and numerous peripherals to reduce environmental impact.	Chou and Chou, 2012
7	Green Digital technology and Digital technology for green	The favorable influence of digital technology on business and economic operations is referred to as the second-order effect. The first-order effect is the negative environmental impact of digital technology development, usage, and disposal. This viewpoint sees information technology as detrimental to environmental sustainability. As a result, Green Digital technology refers to the manufacture, usage, and disposal of digital technology more environmentally friendly.” This viewpoint sees digital technology as a component of eco-sustainability solutions. As a result, utilizing digital technology to make businesses greener is called employing digital technology for greening.	Molla and Abareshi, 2012

### Research gap

Although several studies have investigated the role of digitalization in various economic, social, and environmental factors, most of these studies have provided empirical evidence regarding the developmental, social, and environmental impact of digitalization. Still, these studies are limited in several ways. For instance, the existing studies merely mentioned the detailed or influential channels (a mechanism) through which digitalization affects sustainable development, social development, and environmental quality. Besides, these studies are limited to the investigation of a particular country or region while lacking the generalizability or general overview regarding the influence of digitalization on these three sectors. Following such limitations in the existing literature, this research tends to provide a generalized overview, adoption, implementation, and effective mechanism through which digitalization could be favorable for sustainable development, social development, and environmental sustainability. Since the earlier studies focus on the influence of digitalization in a specific region and a specific sector, i.e., either economic, financial, or environmental. Unlike the existing strand of literature, this study provides a thorough overview of the influence of digitalization on economic, environmental, social, governance, and sustainability sectors.

## Theoretical framework, hypothesis development, and methodology

In the existing literature, three prominent conceptions of digitalization and innovation have been used. All three perspectives envision innovation as a progression of stages across time. The initial notion of “information technology (IT) innovation” refers to the acceptance and dissemination of new IT-enabled procedures, goods, and services by organizations (Fichman, 2004; Jeyaraj et al., 2006). Within this perspective, innovation refers to the acceptance of a previously existing IT artifact by a new organization, likely influenced by multiple technical, organizational, and environmental factors. IT dissemination and integration are concepts connected to innovation in IT.

Another conceptualization of “digital innovation” refers to a product-centric approach in which digital and physical goods are combined to generate advanced products (Yoo et al., 2010; Lee and Berente, 2012). In the under-discussion conception, innovation involves the act of IT artifacts’ underlying architectures in facilitating and restraining the production of advanced IT artifacts, as well as the consequences for organizing and managing innovation inside organizations. Digital innovation is connected to design, but it takes a broader viewpoint than design research and focuses on a broader spectrum of ideas. Lastly, digital innovation refers to the use of IT artifacts inside enterprises that demands considerable transformation and results in the creation of new processes, services, or goods (Swanson, 1994; Fichman et al., 2014). This paradigm includes the technical and organizational components of change related to the creation of new IT-enabled services.

In conclusion, digital innovation encompasses initiating (induces, innovativeness, decision-making), developing (creating, constructing, embracing), integrating (placing, preserving, mentoring, rewards), and exploiting (maximization of returns, maintaining existing systems/data for advance purposes; Cooper and Zmud, 1990) operations. These four processes are not required for all digital innovation initiatives, may occur in any sequence, and may be hard to isolate in reality.

In companies, digital innovation does not emerge in solitude. Digital innovation may be seen as a strategic approach managed and implemented by the IT services division. However, the current organization’s business plans, cultures, and practices may have a substantial influence on digital innovation and serve as a necessary background for digital innovation. This organizational context may influence and be influenced by digital innovation projects (Pentland and Feldman, 2008). Furthermore, digital innovation might alter the company by allowing the development of new business models (Fichman et al., 2014).

Additionally, the competitive environment influences the four digital innovation procedures. For instance, institutional theory (King et al., 1994) and social contagion (Angst et al., 2010) show that enterprises launch digital innovation via processes entrenched in the competitive world. Similarly, digital innovation may alter the competitive landscape in which businesses operate (Vaia et al., 2012).

Apart from the discussion on the conceptualization of digitalization, the latter also influences several sectors of the economy, where environment, social development, and sustainability are highlighted the most in the existing literature. Specifically, Digital technologies have evolved very swiftly than any other breakthrough in human history, altering cultures and reaching almost fifty percent of the emerging world’s population in less than two decades. Technology may be a huge equalizer by boosting connection, access to financial services, accessibility to trade, and public services^2^. Besides, the recent literature also claimed that enhancement in digitalization leads to sustainable development via improving various economic, financial, and trading factors – development of which consequently leads to achieving sustainable development goals of eliminating poverty, increasing health facilities, etc. Based on the above discussion and given literature, this study assumed that digitalization could be advantageous for sustainable development, which is given as: $\delta_{1}=\frac{S\,D_{t}}{D\,i\,g_{t}}>0$. Where *SD* is sustainable development, *Dig* is digitalization, and *t* is the time. Similarly, advancement in digitalization leads to enhanced learning capabilities, learning environment, student output, better job performance, creative thinking, and student innovation (Deng et al., 2022; Wannapiroon and Pimdee, 2022). Also, digitalization favors governance by strengthening the institutional structure through check and balance, the quick implacability of regulations, etc. Therefore, this study assumes that digitalization could also favor social development and governance, given as: $\delta_{2}=\frac{S\,D\,G_{t}}{D\,i\,g_{t}}>0$. Where *SDG* is sustainable development and governance. Moreover, digitalization could also influence environmental quality. For instance, the development of digitalization helps in reducing fossil fuel demand by enhancing green total factor energy efficiency, drives the export value of green goods, promotes green globalization, declines the production scale of pollution-intensive enterprises, and reduces carbon and greenhouse gas emissions (Wen et al., 2021; Gao et al., 2022; Ha and Thanh, 2022; Ma et al., 2022). Therefore, it could be assumed that digitalization may enhance environmental sustainability, expressed as: $\delta_{3}=\frac{E\,n_{t}}{D\,i\,g_{t}}>0$. Where *En* represents environmental sustainability.

After discussing the theoretical framework and hypothesis, this study provides a methodological setup through which the study is conducted. A rigorous selection, a comprehensive review, and synthesizing pertinent literature from the previous two decades have been used to conduct this research, as is the generally accepted method for doing reviews (i.e., Aldieri et al., 2019; Del Río Castro et al., 2021). The assessment is associated with qualitative strategies, taking into account the most pertinent pieces of knowledge from all relevant areas of study, mainly peer-reviewed articles in JCR journals, but also appropriate secondary information, as some of the concepts remain inadequately investigated in the research literature. Existing discussions on sustainable development, the SDGs, and the consolidation between sustainable development and digitalization have been identified, clustered by themes, and critically analyzed in order to create both a big picture and comprehensive perspectives about the reasoning and policy, functional, and research significance of such discourse.

A simultaneous scanning was needed to assess applicable practice-based articles, appropriate measures (e.g., the Global eSustainability Initiative “GeSI”), and Multilateral Organizations (e.g., United Nations Agencies), as well as perspectives generated by the various forums which have been shaping the knowledge base. Once the papers were chosen, a preliminary review of the abstract/executive summary was performed to determine relevancy. The complete texts of relevant papers were then exhaustively evaluated, summarized, and grouped based on their main ideas and research issues.

## Discussion

### Digitalization and sustainable development

Digitization is a game-changer that has altered how people think and shape the world. It has offered firms a new dimension through which to expand. It has aided in bringing people together on a social level and putting them on the same digitization platform. Digitalization is the information flow of objects, sights, and sounds via a quick, spontaneous conduit of impulses. The product is an electronic version of the object, or more accurately, a digital image and a digital form of the signal. Instead of focusing on transactions and systems, consider the various technologies involved in creating value: i.e., smartphones, telemetry, sociodemographic factors, big data, metadata, analytics, behavior, expression, network equipment, and so on. Each is a component of a burgeoning digital economy and a potential source of future wealth. Economic stability is achieved due to cost efficiency and great growth prospects. As a result, our strategy aims for general expansion and brings us closer to long-term development.

In recent years, the manufacturing output of services and goods has changed substantially, expanding swiftly as digital technologies enhance communication throughout the supply chain (Correani et al., 2020). Industry 4.0 refers to the concept that the 4th industrial revolution would depend on digitization rather than automated processes alone, as the third did, and therefore that futuristic manufacturing would be adaptable inside factories comprised of “smart” devices. This approach results from an applications pull requiring the system’s standardization, quick development times, personalization on demand, flexibility, establishing secure and safe processes, resource conservation, and decentralization of decision-making. Likewise, there is a technological drive that will allow for higher mechanization and automation, connectivity of components leading to fully digitalized settings, and a rise in shrinkage (Lasi et al., 2014; Oztemel and Gursev, 2020).

The next wave of digitalization represents new ways of thinking in which we strive to add value and represents the main findings from the existing literature given below:

• People have more choice and independence when they use a lot of knowledge.
• There is a need to develop new methods based on people’s knowledge and capacity to learn more and then implement them effectively to meet their requirements, goals, and ambitions.
• Increasing coordination, resource deployment, and better, faster, and more in-depth decision-making improve operations and performance.
• Digitalization enhances productivity, lowers production costs, increases exports of green products, and promotes green globalization, which not only enhances economic growth but also leads to sustainable development.

Thus, enhancement in digitalization leads to sustainable development, as evident in the extensive existing literature and theory mentioned (Irtyshcheva, 2021; Aleksandrova et al., 2022; Noeh et al., 2022; Novikova et al., 2022; Ren et al., 2022). Hence, the hypothesis – digitalization leads to sustainable development is valid. Further, creating new growth models by combining all competencies in novel ways. We’ve been “Being Digital” for over two decades, and although there’s still a long way to go in terms of digital representation, there are also new prospects outside digital representation that we’re only now beginning to realize (Why digital is the key to Sustainable Development; Gartner blog; The Evolution of Digital Media).

Apart from the earlier discussion, it can be observed that the global spending on the digital transformation of technologies as well as services is increasing over the years.^3^

It is anticipated that in the coming years, the spending on digitalization will further increase throughout the world. Due to the increased investment in this specific sector, the revenue is also expected to rise in the coming years. Thus, digitalization could be used as a tool for enhancing the sustainability of the global economy.

Digital technologies, with their enhanced connectivity and networking, are revolutionizing society, allowing for more communication, services, and trade. While the digital economy may boost productivity and benefit local and global economies, it also raises questions regarding social (i.e., the advantages and costs imposed by disruptive digital technologies on social networks and ways of life, including dangers to economic sustainability and the emergence of economic inequalities) and environmental (i.e., natural resource stewardship and concern for future generations) well-being. Policymakers in national governments and international organizations such as the United Nations and the Organization for Economic Cooperation and Development (OECD) increasingly use digitalization to assess the Brundtland Report’s basic sustainability policy concepts. Various viewpoints on controlling the digitalization process have been expressed, and national governments continue to disagree on the optimal approach for promoting long-term digitalization. The following are our ideas and suggestions:

• Governments should make the appropriate efforts to enhance public understanding of the connections between digitalization and long-term economic growth, and digitalization companies should learn more about their customers’ sustainability choices.
• It is about establishing whether sustainability standards let customers make their judgments about locations, the environment, and resting circumstances. Supporting the sustainability component does not limit digitization.
• Governments and industry stakeholders should explore developing lasting ties between digital and environmental variables to influence tourist growth substantially.
• Digital technology must be familiarized with current findings to discover appropriate digital and environmental aspects that substantially influence economic growth.
• New tools of digitalization that could embrace sustainability should be created. This responsibility applies to governments and tourist sector businesses developing intervention programs or supporting policies.

From an economic aspect, tourism is lauded as a source of income for local communities. Tourism is a hazard to the ecosystem from an ecological aspect. Sustainable tourism should have a minimal detrimental impact on tourist destinations and a good social impact. The tourist economy’s digitalization helps businesses run more efficiently, benefiting customers. The integration of digital technology into the tourist sector, known as digital transformation, leads to major changes in how the world does business, communicates, and evolves nationally and worldwide. Tourism companies may take advantage of new opportunities created by digitization. Technological advancements and customer behaviors are also evolving (Abbasian Fereidouni and Kawa, 2019). Simultaneously, the competition heats up, and businesses must keep up with digitization to stay competitive.

This research intends to contribute to the body of knowledge on challenges affecting the tourism industry by presenting a new general theory on the impact of sustainability and digitization on tourism. The study complements prior research, fills a gap in the literature, and offers a comprehensive theoretical framework for recognizing and assessing the problem of economic growth in the tourist sector, as well as its role in the modern economy, in the context of achieving sustainable development goals (United Nations, 2015). As previously said, we would like to expand on the question: Can digitalization be considered a transformative force in the tourism industry in the age of the internet economy, considering sustainability factors?

### Digitalization and its socio-economic impact

Advances in communication and IT have ushered in a digital revolution that is reshaping how the world works, learns, communicates, and does business. Scientific research has shifted its focus to digitalizing social and economic systems. The “digital economy” concept can be approached in two ways. The first views the information economy as a framework based on the global market for information and communication technology while keeping the fundamental principles of industrial-type economic development, as stated in numerous works by both western and Russian thinkers (Kundishora, 2010). The second strategy is based on the fact that an information economy is fundamentally a new type of activity organization (Dyatlov et al., 2018). Supporters of both methods acknowledge that digitization is a worldwide phenomenon that presents humanity with new possibilities and difficulties (Matveeva and Nikitayev, 2018). Most modern nations include digital technology’s social and economic dangers in their growth goals (Gruber, 2017).

Digital inequality is becoming an important social and economic factor in increasing the digital economy, in addition to differences in the levels of digitization of various areas and sectors of the economy. Due to the digital gap, a population segment has no or limited access to information resources (Castells et al., 2009). As a result, members in this category have limited social opportunities, which harms public production’s economic efficiency, cultural expansion and preservation, and the population’s educational attainment. New societal hazards have emerged because of digital inequality: “The worldwide trend is that the information economy connects those who value it to its network but disables those who have no value for it (thereby further reducing their chances of gaining some value)” (Manyika et al., 2013).

Digital inequality is a systemic problem that impacts all aspects of territory development’s resource potential and necessitates a comprehensive solution. Given the current state of IT development, economic concerns should be considered critical in solving digital inequality. The regulatory consequences of the state and other social institutions should be prioritized. The necessity to limit the influence of economic variables that aggravate digital inequality is linked to economic growth and socio-economic development challenges. The lack of availability of modern digital technologies to the public due to a lack of education required for their development, as well as the difficulties faced by agricultural producers in fully engaging in the production and use of specialized information resources, are examples of digital inequality.

Many aspects of our social environment have been impacted by digitalization. However, we shall focus on the five primary topics discussed in the following sub-sections and also reported.

#### Literacy

According to the corporate responsibility framework for digitalization, digital literacy is one of the thrust areas for empowerment in society. The primary goal of digitalization is to address gender and socio-economic inequities in communities by teaching both men and women digital literacy. Companies should find and pick causes strongly related to their business and philosophical DNA, according to digital technologies, to ensure widespread adoption and implementation of the program. Through education, skills training, or other empowerment programs, we promote digital literacy in our initiatives for both men and women. Computer education support, internet access through IT laboratories, and systematic instruction in important digital skills such as using the internet for information sources and knowledge extension that could also digitally empower women. It is believed that initiatives to pique young girls’ and women’s interest in computers and the internet would boost their employability and improve their overall quality of life. Not only in the gender inequality, instead, digitalization helps increase the efficiency, knowledge, learning ability, multi-tasking, student innovation, and ability to do research in the students, which is also evident in the (Deng et al., 2022; Wannapiroon and Pimdee, 2022; Wekerle et al., 2022). Hence, all these properties further boost student capability for employment, income generation, and better living standards.

#### Governance

Due to digital transformation in governance, all processes and procedures have become more efficient and transparent, as there is no longer any manual interference. Unnecessary delays, bribery, bureaucratic dominance, and corruption have been reduced to the bare minimum. E-governance has provided citizens with a very democratic and open system. It is also easily accessible to the rural populace, allowing them to connect with the emerging globe. E-portals, for example, house all the policies, rules, and regulations. Because everything is tracked digitally, employees must follow them. Employee information, remuneration schemes, taxes norms, labor laws, and regulations are digitally monitored, and attendance is kept online. This has aided in making the entire process more stable and transparent. Looking at it from a global perspective, we may estimate that roughly 2% of GDP is spent on this sector, which could easily be avoided by channeling 1-governance. E-voting systems, e-passports, e-tax filing, and other similar services are extremely cost-effective and save significant time and energy. As a result, there is much room for digital platforms in this area.

#### Health

The healthcare business has a lot of room for digitization because it is one of the fastest-growing and most populous sectors. We’ve created several user-friendly programs that aid in updating, tracking, and planning health-related personnel requirements. Mobius, which interacts with certain health institutes, is one of the applications. It is intended for internal HCL workers and links their requirements and available health care options. This is kept up to date digitally and effectively serves the population of 1.15 million people. Once again, a very cost-effective strategy that has the potential to save up to 12% of gross domestic product (GDP). The IT tsunami has had a tremendous impact on the art and science of medicine around the world, thanks to the digitalization of medical files, now known as electronic health records, and the provision of e-health exams, where online suggestions and talks with physicians are arranged, facilitating sugar, blood, and stress levels, among other things. Only a few digital devices can be used to do a checkup online.

#### Culture

One facet of culture that the digital era effectively addresses is culture. We are all part of a large population that is geographically dispersed. We have all been connected because of the digitalization bridge. This link facilitates cultural interaction and interchange, resulting in a culturally rich and socially functioning society. Digitalization is a worldwide phenomenon that affects every country. Using the internet and digitalized tasks such as online conferencing, e-mails, video calling, etc. Geographical distances have narrowed, and cross-cultural contact links have been developed. The employment of new digital media and technological tools has aided in developing greater cross-cultural encounters and information sharing. The information and communication technology instruments offer cultural preservation, integration, and diversity potential.

#### Environment

There’s no doubting that most digital gadgets or platforms are eco-friendly and have contributed to minimizing carbon emissions. HCL has completely embraced digital in most activities, with all paper publications converted to e-publications. iPads, laptops, and mobile phones can access the reports and data. This method reduces the amount of paper used as well as the expense. Similarly, conducting business online rather than using physical resources has contributed to environmental conservation. It is estimated that 1% of the cost of turning digital in various ways may be saved for a healthier environment.

From the above discussion, it is clear that the social development of a society relies on digital technologies as with the advent of digital technologies, the social developmental indicators enhance. Therefore, the findings are consistent with the existing theory and existing literature (Lavicza et al., 2022). Therefore, the recent studies also force the implementation of digitalization in various educational and governance institutes (Shurygin et al., 2022).

### Role of digital technology in environmental sustainability

Because digital technology greatly influences energy consumption and environmental problems, researchers and practitioners have increasingly focused their attention on achieving energy and environmental sustainability using digital technology. This research model is based on the political-economic framework and examines digital technology’s role in energy and environmental sustainability. At various phases of implementation, factors and outcomes were retrieved. We discovered that digital technology was largely used to monitor and report on an organization’s sustainability impact; nevertheless, the data supplied by these platforms boosted organizational commitment to environmental sustainability and helped these businesses develop sustainable practices. On the other hand, many organizations save to contribute to the depletion of natural resources by consuming more materials and energy than they generate and generating more pollution and waste than the ecosystem can absorb (Dyllick and Hockerts, 2002). Such operations have driven up prices in certain situations and hampered firms’ capacity to satisfy regulatory requirements, such as the governing carbon emissions. On the other hand, organizations that exhibit ecologically responsible behavior strengthen their legitimacy, gain a competitive advantage (Kuo and Dick, 2010), and assure the long-term sustainability of both themselves and their environs (Hart et al., 2003).

The IT sector has also been chastised for having a harmful environmental influence (Urquhart, 2010). Excessive energy usage, greenhouse gas emissions, and hazardous waste disposal are all negative consequences of digital technology systems (Murugesan, 2008). In response to these challenges, smaller and portable systems with lower cooling, energy, and disposal demands, as well as improvements like server virtualization and sensor technologies, have been developed. In addition to these responses, the industry has developed tools for analyzing and evaluating organizational practices, such as the IBM Enterprise Energy Management System (EEMS), which has tracked more than 105 energy conservation programs for 2 years, saving 16,500 MWh of power - worth US$ 1.35 million (Simmonds and Bhattacherjee, 2012). Such operations have driven up prices in certain situations and hampered firms’ capacity to satisfy regulatory requirements, such as those governing carbon emissions. On the other hand, organizations that exhibit ecologically responsible behavior strengthen their legitimacy, gain a competitive advantage (Kuo and Dick, 2010), and assure the long-term sustainability of both themselves and their environs (Hodgin, 2008).

#### Digitalization’s role in climate research

Climate research examines weather conditions and projections using multi-spatial-temporal climatic data as a baseline. The digitization of historical and real-time climate data has transformed extreme weather prediction and the development of prevention and adaptation methods (Munang et al., 2013). This section discusses the importance of digital solutions in attaining sustainability practices and how to understand their capabilities. The use of digitization in biodiversity study, earth observation, and climate action are three main areas of our attention. Human-caused environmental consequences, such as urbanization, pollution, globalization, greenhouse gas emissions, and climate change, have been steadily growing since the Anthropocene period. Quantifying the effect of the Anthropocene based on current evidence is a contentious issue since the question of when the Anthropocene began still looms large (Lewis and Maslin, 2015). Gathering and making electronic data of historical and real-time biodiversity data has grown in popularity throughout the century.

The Global Biodiversity Information Facility (GBIF) is a non-profit organization that provides an online, interconnected network of biodiversity statistics acquired from worldwide biological surveys and collections. The Global Biodiversity Information Facility (GBIF) has almost one billion biodiversity occurrence records, with nearly 150 million based on natural history museum specimens (Yesson et al., 2007). However, according to estimates, only around 10% of specimen samples are available in electronic form (Ball-Damerow et al., 2019). Online public participation can speed up the digitization of biodiversity study specimens by allowing three rounds of transcription, geotagging, and specimen annotation. When citizen science data is used, there are difficulties with data quality. Transcription standardization, geographic data correctness, targeted training, flexible and multistage database management systems, proper statistical methods, and data validation protocols are data quality control strategies that might help overcome this problem (Ellwood et al., 2015). It is undeniable that digitizing collection-based research helps to compile and assess biological baselines for estimating the consequences of climate change, changes in land use (physical and biological qualities of land) and land cover (human use of land), invasive species, and caused by human activity consequences on species diversity (Hedrick et al., 2020).

Some of the outcomes of globalization are given below:

• Enhancing the grid’s capacity to include more volatile renewable energy, developing an interlinked grid with multi-directional energy flow, and extending the utilization of demand response mechanisms are all ways to improve the grid’s integration of renewable energy (i.e., smart charging of electric vehicles).
• Enables electric cars to supply elastic load and storage sources for the electric grid, as well as enhancing fuel economy (e.g., via dynamic routing) and facilitating automated driving technology.
• Energy management systems, mechanical cooling and heating systems, and related equipment and appliances may increase comfort by decreasing energy use in buildings.
• Optimizing resource and energy use, enhancing supply chain operations, and permitting diversification strategy focuses on ecological features.
• Supporting preventive maintenance, identifying and eliminating emission leaks, and enhancing the environmental impact of the oil and gas industry.
• Reducing pollution-intensive inputs (such as fertilizers) and water, enhancing livestock productivity and veterinary medicine, allowing vertical and urban farming, and enhancing carbon sequestration accounting in agriculture.

Where such findings are consistent with the existing literature as well as the persisting theory that illustrates the progressive role of digitalization in environmental sustainability (Garske et al., 2021; Ramos-Meza et al., 2021; Wen et al., 2021; Gao et al., 2022; Ma et al., 2022; Ren et al., 2022).

The Sustainable Development Goals (SDGs’) interconnectedness is evident. Sustainable techniques have shown synergies that can help minimize the consequences of climate change. Thanks to digitalization and artificial intelligence, low-carbon energy systems can monitor and forecast climate and biodiversity changes throughout time with highly efficient renewable energy integration. Multi-spatial-temporal climatic data serve as a foundation for understanding climate variability and future forecasts in climate research. Real-time climate data and the digitalization of historical climate data have transformed forecasting and provided a framework for comprehending current climatic phenomena and their implications for biodiversity. The current use of Internet of Things technology has significantly enhanced data collection methods and real-time big data analysis, which could facilitate implementation. Investigating the long-term effects of climatic and anthropogenic factors on the environment and ecosystems is necessary.

Digitalization is a game-changing weapon for researchers racing against the clock to mitigate climate disasters. By providing solutions and assisting in the construction of a smart Green Planet, digitalization explains the path toward a smart Green Planet. The value of combining big data management and artificial intelligence has been demonstrated. Particular emphasis should be made on the consequences of uneven data access, leading to digital poverty and increasing disparities rather than narrowing them. The cloud should be used to strengthen the cybersecurity of highly networked systems. The benefits of bringing big data into our daily lives, on the other hand, can dramatically improve quality of life while also assisting humanity in tackling long-term challenges such as human, biodiversity, and planet resilience.

## Conclusion and policy implications

A bright spot on the horizon is the development of digital technologies, which has the potential to direct and spark the transformation necessary to achieve all 17 of the Sustainable Development Goals. This research contributes to the growing body of knowledge about the critical role of digitalization – referred to as digital technologies or the internet of things, which have been highlighted for their potential to address major problems in the social-economic and energy nexus, enhance social well-being, and mitigate the effects of climate change. Sustainable techniques have shown synergies that can help minimize the consequences of climate change. Further, it tends to provide a generalized overview, adoption, execution, and effective mechanism that is required for sustainable, social development, and environmental sustainability.

Globally, digital technology is expanding the scope of sustainable agricultural land, energy conservation, resource management, and enhancing the associated productivity, services, and livelihood security. For instance, GIS and remote sensing technology employment have increased agricultural yield via data maps and efficient land use patterns, defining crop varieties and tracking agroecosystem activities. Big data and digitalization play a crucial role in achieving these goals, and our study reflects their experiences and insights. For instance, the study findings asserted that digitalization plays a substantial role in a country’s or region’s sustainable development by enhancing its capabilities toward achieving sustainable development goals of poverty and hunger elimination, provision of health facilities and quality education, industrial innovation, and infrastructure, strong governance, and institutions, tackling environmental hazards, to name a few.

Henceforward, the findings of this study lead to the recommendation of policies that could help both industrialized and emerging economies to achieve sustainable development. First, government operations must limit their impact on the environment. there must be some concrete measures for agencies as environmental standards that aid in incorporating sustainability in management procurements. With proper digital tools, a circular economy can be created that will radically limit emissions and the promotion of green and renewable technologies can be encouraged. Specifically, the global economies are required to revise policies by considering digital technologies and innovation in the industrial, agriculture, social, and climate-related sectors. The construction and implementation of digitalized policies are hardly possible without paying proper attention to the authorities and investment in the digital technology, innovation, and research and development sectors. Following the outcomes of the study, the global economies need to encourage digitalization at school or college levels, which will help the students as well as the general public to recognize the importance and efficient utilization of digitalization for educational, income, and learning, or ease of doing business purposes. Moreover, the findings asserted that digitalization could play a substantial role in governance quality. Therefore, this study recommends the construction of policies that could consider a digital setup in the institutional as well as governance sectors. Consequently, this will lead to promote political and bureaucratic accountability, increase check and balance, and reduce corruption in the country. Hence, following such policies could lead the economy to achieve social development, environmental sustainability, and sustainable development.

Although this study covers wide-ranging literature to analyze the importance of digitalization in sustainable development, environment, and social development, this study is still limited in delivering empirical insights via estimation of the data. Therefore, this study suggested that future researchers empirically analyze digitalization’s influence on various economic, environmental, and social factors and indicators. Another limitation of this study is that it focuses on the available literature, which extensively analyzes developed regions. Therefore, future researchers are directed to analyze emerging as well as under-developed economies for the relevant policy implications. Various parametric approaches such as linear regressions and non-parametric approaches such as quantile regression and method of moment quantile regression could be used to explore the nexus more comprehensively. Moreover, future studies could also use ARDL approaches for the identification of the short and long-run estimations.

## Data availability statement

The original contributions presented in this study are included in the article/supplementary material, further inquiries can be directed to the corresponding author/s.

## Author contributions

JX: concept, literature review, and review. SS: data processing, theory, and editing. WL: discussion, conclusion, concepts, and policies. All authors contributed to the article and approved the submitted version.

## Footnotes

1
www.openstreetmap.org

2 See https://www.un.org/en/un75/impact-digital-technologies#:$nsim$:text~=Digital%20technologies%20have%20-advanced%20more,can%20be%20a%20great%20equaliser.

3
https://www.statista.com/statistics/870924/worldwide-digital-transformation-market-size/
